# Enjoyed His Chills

**Published:** 1906-12

**Authors:** 


ENJOYED HIS CHILLS.—Down in certain sections of the Mississippi river bottoms there is such an air of unconcern that the first thought of a traveler is: "These people are too lazy to entertain a hope." It is, however, not wholly a condition of laziness that produces such an appearance of indolence. Laziness may play its part, and, moreover, may play it well, but it can not hope to assume the leading rôle. What, then, is the principal actor? Chills. There are men in those bottoms who were born with a chill and who have never shaken it off.

Some time ago, while riding through the Muscadine neighborhood, I came upon a man sitting on a log near the roadside. He was sallow and lean, with sharp, knob cheek-bones, and with hair like soiled cotton. The day was intensely hot, but he sat in the sun, although near him a tangled grape-vine cast a most inviting shade.

"Good morning," said I, reining up.

"Hi."

"You live here, I suppose?"

"Jest about."

"Why don't you sit over there in the shade?"

"Will, when the time comes."

"What do you mean by 'when the time comes'?"

"When the fever comes on."

"Having chills, are you?"

"Sorter."

"How long have you had them?"

"Forty-odd year."

"How old are you?"

"Forty-odd year."

"Been shaking all your life, eh?"

"Only half my life; fever on the other half."

"Why don't you move away from here?"

### Atonic Indigestion

A large proportion of all cases of indigestion are the result of atony of the stomach and intestinal walls.

### Gray's Glycerine Tonic has a unique value in this particular condition as it rapidly **restores muscular vigor, increases secretory activity** and **checks fermentation.** As a consequence **the results are permanent—not transitory.**

**The Purdue Frederick Co.,**
**298 Broadway, New York.**

"Because I've lived here so long that I'm afeared I might not have good health nowhere else."

"Gracious alive! do you mean to say that having chills all the time is good health?"

"Wall, health mout be wuss. Old Nat Sarver moved up in the hills some time ago, was tuck down putty soon with some new sort of disease and didn't live more'n a week. Don't b'lieve in swappin' off suthin' that I'm used to, for suthin' I don't know nothin' about. Old-fashion, every-day chiils air good enough for me. Some folks, when they git a little up in the world, mout want to put on airs with dyspepsia and bronckichus, and glanders, and catarrh, but, as I 'lowed to my wife, old chills and fever war high enough fur us yit a while. A chill may have drawbacks, but it has its enjoyments, too."

"I don't see how a chill can be enjoyable."

"Jest owin' to how you air raised. When I have a chill it does me a power o' good to stretch, and I tell you, that a fust-rate stretch when a feller is in the humor ain't to be sneezed at. High-o-hoo," he gaped, threw out his legs, threw back his arms and stretched himself across the log. "It's sorter like the itch," he went on. "The itch has its drawbacks, but what a power of good it does a man to scratch. Wall, my fever is comin' on now, and I reckon I'll hurry and git up thar under the shade."

He moved into the shade and stretched himself again.

"How long will your fever last?" I asked.

"Wall, I don't know exactly; three hours mebby."

"Then what?"

"Wall, I'll funter around a while, chop up a little wood to get a bite to eat with, swap a hoss with some feller, mebby, and then fix myself for another chill."

"Have you much of a family?"

"Wife and grown son. He's about the ablest chiller in the county: w'y, when he's got a rale good chill on, he can take hold of a tree and shake off green persimmons. Wall, have you got to go?"

"Yes."

"Good-bye, then. When you git tired livin' up thar among them new-fangled diseases, come down here whar everything is old-fashioned, comfortable and honest."—*Opie Read.*

